# When Can an Emergency CTA Be Dispensed with for TIA Patients?

**DOI:** 10.3390/jcm11195686

**Published:** 2022-09-26

**Authors:** Jens-Christian Altenbernd, Razvan Gramada, Eugen Kessler, Jakob Skatulla, Eduard Geppert, Jens Eyding, Hannes Nordmeyer

**Affiliations:** 1 Institute of Diagnostic and Interventional Radiology and Neuroradiology, University Hospital Essen, 45147 Essen, Germany; 2 Department of Radiology and Neuroradiology, Gemeinschaftskrankenhaus Herdecke, 58313 Herdecke, Germany; 3 Department of Neurology, Gemeinschaftskrankenhaus Herdecke, 58313 Herdecke, Germany; 4 Radprax MVZ Nordrhein GmbH Department of Interventional Neuroradiology, St. Lukas-Klinik, 42697 Solingen, Germany; 5 School of Medicine, Department of Health, Witten/Herdecke University, 58455 Witten, Germany

**Keywords:** CTA, TIA, stroke

## Abstract

Background: Transient ischemic attacks (TIAs) and minor strokes are often precursors of a major stroke. Therefore, diagnostic work-up of the TIA is essential to reduce the patient’s risk of further ischemic events. Purpose: With the help of this retrospective study, we aim to determine for which TIA patients a CT angiography (CTA) is not immediately necessary in order to reduce radiation exposure and nephrotoxicity. Material and Methods: Clinical and imaging data from patients who presented as an emergency case with a suspected diagnosis of TIA at a teaching hospital between January 2016 and December 2021 were evaluated. The included 1526 patients were divided into two groups—group 1, with major pathologic vascular findings in the CTA, and group 2, with minor vascular pathologies. Results: Out of 1821 patients with suspected TIA on admission, 1526 met the inclusion criteria. In total, 336 (22%) had major vascular pathologies on CTA, and 1190 (78%) were unremarkable. The majority of patients with major vascular pathologies were male and had a history of arterial hypertension, coronary heart disease, myocardial infarction, ischemic stroke, TIA, atherosclerotic peripheral vascular disease, smoking, antiplatelet medication, had a lower duration of TIA symptoms, and had lower ABCD2 scores. Conclusions: We were able to demonstrate a direct correlation between major CTA pathologies and a history of smoking, age, hyperlipidemia, history of peripheral arterial disease, and a history of stroke and TIA. We were able to prove that the ABCD2 score is even reciprocal to CTA pathology. This means that TIA patients without described risk factors do not immediately require a CTA and could be clarified in the course of treatment with ultrasound or MRI.

## 1. Introduction

Transient ischemic attacks and minor strokes are often precursors of a major stroke [1]. Studies have shown that patients with a transient ischemic attack (TIA) have an increased risk of suffering a stroke in the following 3 months. The first 48 h after the TIA are particularly risky [2,3].

Therefore, diagnostic work-up of the TIA is essential to reduce the patient’s risk of further ischemic events [4]. Non-contrast head CT is more readily available than MRI, cheaper, faster, and provides a good depiction of bleedings, although infarcts are better detected with MRI [5,6,7,8]. According to current guidelines, cross-sectional imaging should be performed within 25 min in order to be able to initiate appropriate therapy quickly [9]. A computed tomography of the head without contrast medium within 3 h after the onset of symptoms has a sensitivity of 47–53% and a specificity of 80% with regard to the presence of an acute ischemic stroke [10,11]. Severely affected patients should have standard CT or MRI imaging of the cerebrospinal arteries to assess the indication for mechanical thrombectomy. Ideally, additional perfusion imaging should be performed so that potentially salvageable tissue can be quantified [12,13,14,15,16,17,18]. The CTA has a very high specificity (82–100%) and sensitivity (92–100%) in detecting large proximal cerebral artery occlusions [19]. In addition to perfusion imaging, the collateral vessels can be displayed as a possible prognostic factor and decision making aid via a time-resolved CT imaging [4,16].

Performing CTA has become established in TIA in order to detect vascular pathologies [20]. Recent studies have shown that relevant vascular pathologies are detected by CTA in around 20 percent of TIA patients, meaning that 80% of patients receive an unnecessary CTA [21]. With the help of this retrospective study, we would like to find out for which TIA patients a CTA is immediately indicated or for whom an ultrasound or MRI are sufficient in the course of treatment in order to avoid unnecessary radiation exposure and nephrotoxicity.

## 2. Material and Methods

**Figure 1:**
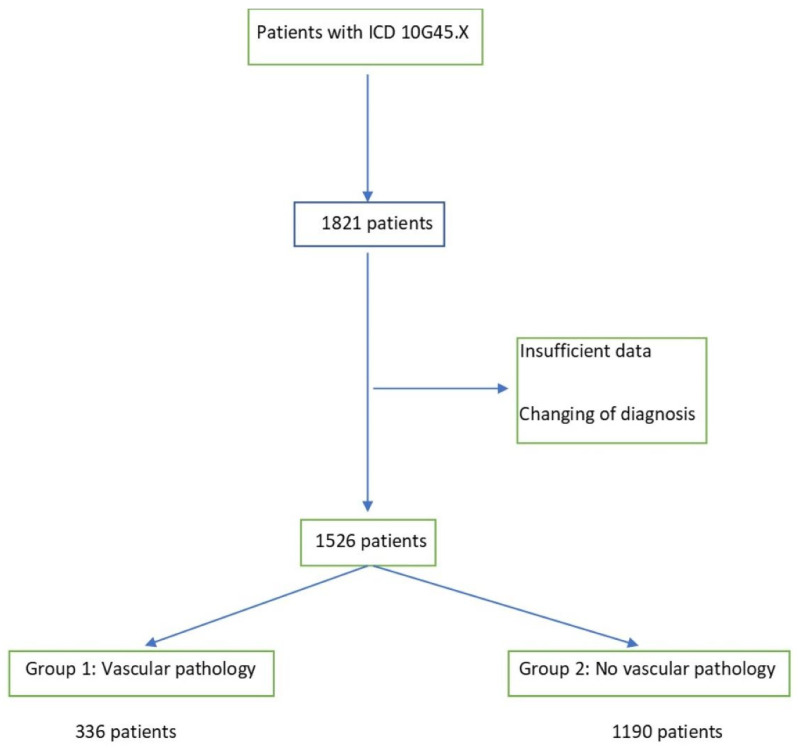
Inclusion flow chart (ICD International Statistical Classification of Diseases and Related Health Problems).

With this retrospective study, clinical and imaging data from patients who presented as an emergency with a suspected diagnosis of TIA at a teaching hospital between January 2016 and December 2021 were evaluated. Out of a total of 1821 patients, 293 patients were not included in the study because of insufficient data or a change in their primary TIA diagnosis during the hospital stay. The included 1526 patients were divided into two groups (Figure 1), with group 1 having major vascular pathologies in the CTA, and group 2 having minor vascular pathologies. Stenoses > 50% (NASCET), occlusions, and dissections were classified as major vascular pathologies, as well as thrombus material within the arteries. According to the North American Symptomatic Carotid Endarterectomy Trial (NASCET), vascular pathologies were considered minor if they lead to a stenosis less than 50% of the vessel [22], vascular pathologies with a stenosis greater than 50% were considered as severe pathologies. In the case of stenoses greater than 50%, vessel dissections, occlusions, and intra-arterial thrombi, the patients were assigned to the first group with ipsilateral vascular pathologies.

In addition to CTA, all patients underwent neurovascular ultrasound performed by experienced neurologists in the emergency department. CTA was reviewed by experienced neuroradiologists.

The image data were collected based on the consensus of two neuroradiologists. The clinical and anamnestic data were taken from the hospital’s internal information system (ORBIS, AGFA) (Table 1).

SPSS 21 was used for statistical analysis (SPSS Statistics, Armonk, NY, USA).

The groups were evaluated with *t*-tests, Wilcoxon–Mann–Whitney tests, and χ^2^ tests.

## 3. Results

We included 1526 TIA patients in this study who received a CTA on the day of admission. Their baseline characteristics are provided in Table 1. The following major pathologies were found: vessel occlusion 14/336 (4%), stenosis > 50% (NASCET) 297/336 (88%), vessel dissection 12/336 (4%), and thrombus 13/336 (4%) (Table A1). TIA patients with major vascular pathologies were predominantly male (68 % vs. 51%, *p* = 0.028), and had a history of arterial hypertension (85% vs. 65%, *p* < 0.001), coronary heart disease (27% vs. 12%, *p* = 0.001), myocardial infarction (19% vs. 12%, *p* < 0.016), ischemic stroke (29% vs. 20%, *p* = 0.013), TIA (34% vs. 21%, *p* = 0.021), atherosclerotic peripheral vascular disease (21% vs. 11%, *p* < 0.001), smoking (32% vs. 18%, *p* = 0.012), antiplatelet medication (61% vs. 38%, *p* < 0.001), a lower duration of TIA symptoms (45% vs. 59%, *p* < 0.001), and a lower ABCD2 score (4 vs. 5, *p* = 0.022).

## 4. Discussion

In this study, we investigated the question of which TIA patients may or may not require relevant CTA pathology testing. A high-grade pathological CTA sometimes has direct therapeutic/interventional consequences and an avoidable normal CTA means unnecessary radiation exposure and potential risks from the contrast agent.

Consistently with other studies, we were able to confirm that gender, duration of TIA symptoms, known hypertension, known coronary artery disease, a history of myocardial ischemia, and antiplatelet medication were significantly associated with relevant pathologies in the CTA, and these could therefore be used to select patients for CTA [4,21,23,24,25,26].

As other studies suggest, we were able to prove that the ABCD2 score has no correlation to CTA pathology, i.e., higher scores were calculated for patients with unremarkable CTA, which may be related to the different assessment of the duration of symptoms undertaken during the calculation of the score [24,27,28,29,30,31,32,33,34]. So, we think the ABCD2 score as a risk stratification has largely become defunct now. It is no longer recommended as a risk stratification tool to be used in isolation, especially since patients with low ABCD2 score who are apparently low risk, could still have significant stroke risk factors.

In contrast to other studies, we were able to demonstrate that major CTA pathologies were observed more frequently in patients with a history of smoking, a higher age, hyperlipidemia, a history of peripheral arterial disease, and a history of stroke and TIA [20,35,36,37,38] with a respective statistical significance. In our opinion, the patient’s risk profile should be considered when deciding whether or not to perform a CTA in patients with TIA.

Patients with carotid stenosis often show a short duration of symptoms, typically with a subtype of TIA, the amaurosis fugax [21,39,40].

A short duration of symptoms therefore indicates a macroangiopathic disease, which should therefore be clarified by means of appropriate imaging [41].

Computed tomography plays a crucial role in the acute diagnosis of transient ischemic attacks—on the one hand, to assess infarction demarcations or rule out bleeding, and on the other hand, to image the arteries supplying the brain using CT angiography. An ultrasound examination of the arteries is too time-consuming in the acute situation and is highly dependent on the examiner. An MRI takes longer and is usually not regularly available 24/7.

There are other benefits to performing a CTA at the front door. This may include more intensive initial antiplatelet therapy—for example, with dual antiplatelet. Therefore, performing a CTA may allow clinicians to give more optimal medical treatment while surgical intervention is being considered.

Performing an early CTA may also allow earlier discharge, since vascular imaging has already been performed and patients do not need to wait for a carotid US.

CTA may change management—for example, if a patient is a likely surgical candidate. This could be another possible criterion that could be used when deciding whether to do a CTA for TIA patients.

Regarding the limitations of our study, it must be stated that, based on previous studies, we made a distinction between more or less than 50% stenosing pathology in the internal carotid artery. Of course, there are arguments for defining the percentage limit differently. In addition, this is only a retrospective study, and prospective approaches could also take into account the direct follow-up of the patients with regard to morbidity, mortality, and necessary interventional or surgical therapies.

In conclusion, we were able to demonstrate a direct correlation between major CTA pathologies and a history of smoking, age, hyperlipidemia, a history of peripheral arterial disease, and a history of stroke and TIA. The ABCD2 score does not help in deciding whether or not to conduct a CTA. This suggests that TIA patients without described risk factors do not immediately need a CTA, and their condition could be clarified in the course of treatment with ultrasound or MRI.

## Figures and Tables

**Table 1:** Characteristics of patients with and without relevant vascular pathology in terms of gender, age, previous illnesses, risk factors, anamnesis, and medication. (NIHSS National Institutes of Health Stroke Scale; DOAK Direct Oral Anticoagulation; TIA Transient Ischaemic Attack; ABCD Age, Blood pressure, Clinical features, Duration of TIA, and presence of Diabetes).

	Major Pathology *n* = 336 (22%)	Minor Pathology *n* = 1190 (78%)	*p*-Value
Age, years ± SD	72 ± 8	63 ± 9	0.011
Sex (male, %)	68%	51%	0.028
NIHSS	0	0	0.729
Duration of symptoms >60 min	45%	59%	<0.001
Arterial hypertension	85%	65%	<0.001
Hyperlipidemia	75%	61%	<0.001
Diabetes mellitus	27%	24%	0.734
Coronary heart disease	27%	12%	0.001
Obesity	22%	19%	0.243
History of myocardial infarction	19%	12%	0.016
Atrial fibrillation	23%	24%	0.422
History of Atherosclerotic peripheral vascular disease	21%	11%	<0.001
History of smoking	32%	18%	0.013
Antiplatelet medication	61%	38%	<0.001
DOAK	7%	8%	0.192
Marcumar	10%	9%	0.433
History of stroke	29%	18%	0.013
History of TIA	34%	21%	0.021
ABCD2	4 (3–5)	5 (4–6)	0.022

## Data Availability

All data relevant to the study are included in the article.

## Appendix A

**Table A1:** Pathology (NASCET North American Symptomatic Carotid Endarterectomy Trial).

	Pathology *n* = 336 (22%)
Vessel occlusion	14
Stenosis > 50% (NASCET)	297
Vessel Dissection	12
Thrombus	13
